# Supplementary material for: The influence of lazy information disclosure on stock price crash risk: Empirical evidence from China
Source: PLoS One. 2023 Aug 18;18(8):e0288552. doi: 10.1371/journal.pone.0288552 (PMC10437866; doi:10.1371/journal.pone.0288552)
Supplement: Attachment — Submitted filename: Review Comments-01.docx [file pone.0288552.s002.docx]

This paper discusses the economic consequences of lazy annual report information disclosure, which has certain novelty.

The following aspects are for the author to further improve the paper reference:

**#Q1**  The article does not clearly explain why lazy information disclosure exacerbated the stock price crash. In 3.1 Lazy information disclosure and stock price crash risk, the author explains that lazy information reduces information content and increases stock price synchronicity, which is acceptable. However, this paper does not test the influence of lazy information disclosure on stock price synchronicity, nor does it use stock price synchronicity as an intermediate variable to test stock price crash risk.

In addition, the author emphasizes in the article: *When the stock index is in the rising cycle, the synchronicity of stock price makes the stock price overvalued, and the stock price is inconsistent with the actual value of the company. When the stock index is in a downward cycle, the synchronicity of stock price will lower the stock price, and the management tends to release false news to maintain the stock price, so that the stock price deviates from the actual value of the company and increases the possibility of stock price crash*.

The contents emphasized above are not controlled and demonstrated in the empirical part, which is too subjective and far-fetched.

Therefore, it is suggested that the author consider the direct impact of Lazy information disclosure on stock price crash risk from the classical hypothesis of stock price crash risk itself. For example, can we consider the hypothesis of managing bad news hiding put forward by Jin and Myesr(2006) and clearly demonstrate the possible explanation of Lazy information disclosure for bad news hiding?

**#Q2** In part *3.2 The regulating role of audit institutions*, the paper also puts forward "*the quality of the annual report information*". The following three details are not clear. This hypothesis is also not strongly supported.

**#Q2.1** The article also did not clearly account for the relationship between Lazy information disclosure and the quality of the annual report information.

**#Q2.2** If big auditors can improve "the quality of the annual report information", then why do companies audited by big auditors still have "Lazy information disclosure"?

**#Q2.3** How the "Lazy information disclosure" after audit by a large auditors differs from other samples.

**#Q3**  *4.2.2 Explain variables* Whether there are relevant literatures for reference.

**#Q4**  This paper tests whether it is appropriate for the relevant variables of financial report to lag 2 periods in the model, and it is common to lag 1 period.
